# Study of psychological state of cancer patients undergoing radiation therapy during novel coronavirus outbreak and effects of nursing intervention

**DOI:** 10.1002/prm2.12026

**Published:** 2020-11-26

**Authors:** Shuchang Lou, Dejing Xu, Xiaodong Li, Yan Huan, Jun Li

**Affiliations:** ^1^ Department of Radiotherapy Department Jiangsu Cancer Hospital, Jiangsu Institute of Cancer Research & The Affiliated Cancer Hospital of Nanjing Medical University Nanjing China

**Keywords:** cancer, COVID‐19, psychological state, nursing, intervention

## Abstract

**Objective:**

To investigate psychological state of hospitalized cancer patients undergoing radiation therapy and evaluate effects of customized psychological intervention on patients' psychological state during novel Coronavirus (COVID‐19) outbreak.

**Method:**

Fifty‐eight hospitalized head and neck cancer patients undergoing radiation therapy were included and received online and offline psychological intervention. General information questionnaire and the Self‐Report Symptom Inventory, Symptom Check‐List90 (SCL‐90) were utilized to investigate and analyze psychological state of hospitalized head and neck cancer patients undergoing radiation therapy before and after intervention. Self‐Rating Depression Scale (SDS) and Self‐Rating Anxiety Scale (SAS) were used to evaluate depression severity and anxiety severity of them.

**Results:**

Overall psychological state of patients (include nine symptom dimensions: somatization, obsessive, interpersonal sensitivity, depression, anxiety, hostility, phobic anxiety, paranoid ideation, and psychoticism) was improved significantly after intervention (*P* < .05). Moreover, scores of SAS and SDS were lowered.

**Conclusion:**

Customized psychological intervention helped to improve overall psychological state of hospitalized cancer patients undergoing radiation therapy during COVID‐19 outbreak and showed encouraging effects on reduction severity of depression and anxiety.

## 1 INTRODUCTION

Since novel coronavirus (COVID‐19) was first described in 2019, the number of COVID‐19 cases has been rapidly increasing and has become a serious threat to human health worldwide.^1^, ^2^, ^3^ Concern for the influence of COVID‐19 on cancer patients has grown as sweeping infection rates continue to multiply. As reported in the guidelines for prevention or control of hospital infections, isolation has significant adverse effects on the behavior, mental health, satisfaction, and safety of inpatients.^4^ Hospitalized cancer patients are recommended to take more thorough protection measures than general population on account of their high risk for developing serious illness from COVID‐19. Such measures include self‐isolation, even from members of their own household.^5^, ^6^ In this critical period, hospitalized cancer patients may be adversely affected by the stressful events of “COVID‐19 outbreak,” “self‐isolation,” and “social distancing,” and this may aggravate pre‐existing psychological distress such as anxiety and depression.^7^ This fact may cause cancer patients to experience increased psychological burden and result in a series of detrimental emotional states. Therefore, cancer and COVID‐19, present a doubled challenge for medical staff, as patients' stable psychological state is essential for ensuring that patients can receive effective and regular cancer treatment. Thus, the psychological state of head and neck cancer inpatients of the Radiotherapy Department of Jiangsu Cancer Hospital were investigated during COVID‐19 outbreak and the effects of the psychological intervention are elucidated in the following article.

## 2 PARTICIPANTS AND METHODS

### 2.1 Participants

Hospitalized head and neck cancer patients in Radiotherapy Department of Jiangsu Cancer Hospital from February 3, 2020 to February 24, 2020 were enrolled. Inclusion criteria: (1) 18 to 75 years old; (2) Elementary school education or above; (3) No major life events occurred during hospitalization; (4) With newly diagnosed head and neck cancer (stages II, III, or IV at diagnosis) (5) Currently in a stable physical condition; and (6) All patients voluntarily participated in the research. Exclusion criteria: (1) Mental and/or cognitive illness; and (2) Reading comprehension and/or communication disorders. All patients and their families were informed and signed informed consent and confidentiality agreement.

### 2.2 Research method

#### 2.2.1 Establishment of research group

Research group included two researchers, one research assistants, and four psychology nurse specialists as intervention nurses. Purpose and significance of this research were introduced and explained. Also, the use and precautions of Symptom Check‐List90, Self‐Rating Depression Scale and Self‐Rating Anxiety Scale were explained. Researchers and research assistants were responsible for the screening and communication of enrolled patients. Research assistants and intervention nurses were responsible for distribution and recollection of the questionnaires, and for implementation of psychological intervention.

#### 2.2.2 Intervention method

*Guidelines of Psychological Adjustment During Novel Coronavirus Outbreak* released by National Health Commission of the People's Republic of China in February 2020 was used as the main guidance in this research.^8^ *Clinical Practice Guidelines in Oncology*, *Version 1*, *2020* published by US National Comprehensive Cancer Network was used as a supplementary guidelines.^9^ Psychological intervention strategies were developed from both guidelines comprehensively which include information sharing, recall of happy/positive events, and emotional catharsis.

Considering safety of hospitalized cancer patients undergoing radiotherapy during COVID‐19 outbreak, and the effectiveness of psychological intervention, online and offline psychological interventions were adopted in this research as recommended in *Psychological Intervention Guidelines During Novel Coronavirus Outbreak*.^10^

Intervention measures include:

1. *Online*: pandemic information was shared in real time through WeChat communication channels; stress‐reducing techniques and activities were taught; patients were encouraged to recall and describe personal experiences of gratitude, happiness, or sense of self‐achievement.
2. *Offline*: through one‐to‐one interview, patients were encouraged to talk about their concerns and worries with intervention nurses in the counseling office (a closed but safe environment). See Table 1 for detailed intervention strategies.

**TABLE 1:** Psychological intervention for hospitalized cancer patients undergoing radiotherapy during COVID‐19 outbreak

Content	Form	Concrete measure	Objective
Information sharing	Online sharing and discussion	WeChat channel “antiCovid‐19 & anti‐cancer” was established to provide continuous information support for patients. Including timely and accurate epidemic news, protection knowledge, typical symptoms, diet, and nutrition precautions for cancer patients, and progress in the treatment. Patients were encouraged to participate in the discussion. Focus on the accuracy of the news obtained by patients, patient's demand for other information, and their response to the existing information.	To meet the needs of patients for information and communication, and to eliminate the uncertainty and panic caused by lack of rational thinking
Stress‐reducing techniques and activities	Online sharing and discussion	Through WeChat, contents of beautiful‐essay, music, and video were shared. Patients were guided to keep busy and habits were developed by reading books and newspapers, practicing Qigong, meditation, writing diaries, listening to radio and music, muscle relaxation training, and other activities.	To help patients to avoid unnecessary concerns and worries and to achieve the goal of emotion stability
Regain of gratitude, happiness, and sense of achievement	Online sharing and discussion	Through WeChat, patients were encouraged to recall, share and describe their personal experiences of gratitude, happiness or sense of achievement. Also, to tell their families about their true feelings. Patients were taught to actively recall such events when they are depressed.	Through recalling, retelling, and sharing experience that is positive, grateful, happy, or can bring sense of achievement to help patients to build confidence, retrieve hope and happiness
Relieve bad mood	Offline one‐to one interview	Through one‐to one offline interview with psychology nurse specialists, patients were encouraged to freely express their worries, concerns, struggles and other emotions in the counseling room. Understandings and support for the patients were expressed by psychology nurse specialists and potential solutions were discussed.	While protecting the privacy and maintaining the dignity of patients, they were allowed to vent their negative emotions, encouraged to face the difficulties, and accept imperfections in their lives. From the perspective of patients, their psychological demands should be understood, and personalized and effective intervention need to be carried out.

#### 2.2.3 Assessment

##### General information questionnaire

According to the purpose of this study, a questionnaire was designed by researchers to investigate general information of patients include age, gender, marital status, and education level.

##### Symptom checklist 90

Current version of *Self‐Report Symptom Inventory* (*Symptom Checklist 90*, SCL‐90) was developed by Delogatis in 1973 from *Cornell Medical Index*. The scale has been proved to have good reliability and validity, as well as adequate sensitivity.^11^ SCL‐90 includes 90 items to evaluate the symptoms of nine main dimensions by using a 5‐point rating scale, including somatization, obsession, interpersonal sensitivity, depression, anxiety, hostility, phobic anxiety, paranoid ideation, and psychoticism. During the administration, it is important to make sure that patients are fully understand the content of the checklist, then carefully read and rate each of the items. The total score is the sum of the items.^12^, ^13^, ^14^

##### Self‐rating depression scale and self‐rating anxiety scale

Self‐Rating Depression Scale (SDS) and Self‐Rating Anxiety Scale (SAS) were developed by Zung in 1965 and 1971 and revised several times. Both SDS and SAS were proved to have good reliability and validity.^15^, ^16^ Both scales include 20 items and each item can be rated with a 4‐point scale from 1 to 4. The total raw score of 20 items in each scale need to be multiplied by 1.25 to get an index score. In SDS, a score lower than 53 indicates no depression, a score from 53 to 62 indicates mild depression, a score from 63 to 72 indicates moderate depression, a score higher than 72 indicates severe depression. In SAS, a score lower than 50 indicates no anxiety, a score from 50 to 59 indicates mild anxiety, a score from 60 to 69 indicates moderate anxiety, and a score higher than 90 indicates severe anxiety.^13^, ^15^

### 2.3 Data collection

Patients who meet the inclusion criteria were asked to complete questionnaires before and after the psychological intervention. Before filling in the questionnaire, uniform instructions were given. After the questionnaire was sent out, the research assistants left and patients were allowed to respond the questionnaire independently. Patients were encouraged to complete questionnaires within 1 hour. Two patients requested orally assistance from families to read questionnaire for them because of fatigue. One day before intervention, 60 valid questionnaires were collected. One day after psychological intervention, data were collected by sending out paper‐based questionnaire, electronic questionnaire through WeChat, or through telephone follow‐ups. In order to ensure the accuracy and validity of the questionnaire, contents of questionnaire were screened by members of research team immediately and clarify ambiguities if there was any. In the process of research, one patient refused to continue to participate and one patient could not be reached. Therefore, there were two dropouts in this research, completion rate was 96.67%.

### 2.4 Statistical methods

The SPSS 22.0 software was used to analyze data. Categorical data were described by frequency and percentage; measurement data were described by mean ± SD, and *t*‐test was used for group comparison. Changes in psychological state of hospitalized cancer patients were investigated by deviation analysis. The difference was statistically significant when *P* < 0.05.

## 3 RESULT

### 3.1 Patient characteristics

A total of 58 hospitalized head and neck cancer patients undergoing radiotherapy during COVID‐19 outbreak were enrolled in this research, with an age of 26 to 72 (46 ± 8.3).

Among all participants, 35 were male and 23 were female. Five were unmarried, 47 were married, and 6 were divorced/widowed. Nine of them attended elementary school or middle school, 22 of them had high school diploma, 17 of them were community college graduates, and 10 of them had bachelor diploma or above.

### 3.2 Comparison of SCL‐90 scores of patients before and after psychological intervention with Chinese national norm^13^

SCL‐90 was performed on head and neck cancer patients undergoing radiotherapy during COVID‐19 outbreak before and after psychological intervention. SCL‐90 scores were compared. The difference in SCL‐90 scores was statistically significant (*P* < 0.05), as shown in Table 2. Improvements in all nine dimensions were observed on head and neck cancer patients undergoing radiotherapy during COVID‐19 which means cancer patients generally have psychological distress during this outbreak and effectiveness of psychological intervention was proved.

**TABLE 2:** Comparison of SCL‐90 scores of cancer patients before and after psychological intervention with Chinese national norm (^−^x ± s)

Item	Before intervention (n = 58)	After intervention (n = 58)	*t* value	*P* value	National norm^13^ (n = 1388)
Somatization	2.12 ± 0.34	1.94 ± 0.33	2.676	.010	1.37 ± 0.48
Obsession	2.06 ± 0.42	1.78 ± 0.52	3.023	.004	1.62 ± 0.58
Interpersonal sensitivity	2.26 ± 0.38	1.84 ± 0.35	5.932	.000	1.65 ± 0.61
Depression	2.51 ± 0.52	2.19 ± 0.48	3.395	.001	1.50 ± 0.59
Anxiety	2.66 ± 0.60	2.13 ± 0.40	6.051	.000	1.39 ± 0.43
Hostility	2.10 ± 0.37	1.75 ± 0.32	5.208	.000	1.46 ± 0.55
Phobic anxiety	2.11 ± 0.52	1.88 ± 0.38	2.745	.008	1.23 ± 0.41
Paranoid ideation	2.07 ± 0.54	1.70 ± 0.45	4.008	.000	1.43 ± 0.57
Psychoticism	1.93 ± 0.39	1.64 ± 0.49	3.488	.001	1.29 ± 0.42
Positive symptom total	41.59 ± 11.32	29.65 ± 6.06	6.657	.000	24.9 ± 18.41

### 3.3 Changes in scores of anxiety and depression before and after psychological intervention

SAS total index scores and SDS total index scores of head and neck cancer patients undergoing radiotherapy during COVID‐19 outbreak before and after intervention were compared. As shown in Table 3, a decreased average SDS score (from 69.07 to 64.83) was observed after intervention. Similarly, average SAS score decreased from 65.03 (moderate anxiety) to 57.81 (mild anxiety) after intervention.

**TABLE 3:** SAS score and SDS score of head and neck cancer patients undergoing radiotherapy during COVID‐19 outbreak before and after intervention

Scale	Before intervention (n = 58)	After intervention (n = 58)	*t* value	*P* value
SDS	69.07 ± 5.30	64.83 ± 5.92	9.288	.010
SAS	65.03 ± 7.13	57.81 ± 7.38	4.771	.008

### 3.4 Changes in severity of anxiety and depression before and after psychological intervention

Changes in severity of anxiety and depression of head and neck cancer patients undergoing radiotherapy during COVID‐19 outbreak before and after psychological intervention were compared as shown in Table 4. Then, 37.9% of patients suffered from severe anxiety before intervention and only 3.4% of patients were in the state of severe anxiety after intervention. In terms of depression, there was 96.7% of patients were severe/moderate depressed and that percentage decreased to 84.4% after intervention.

**TABLE 4:** Severity of anxiety and depression of head and neck cancer patients undergoing radiotherapy during COVID‐19 outbreak before and after intervention

Symptom		Score	Before intervention (n = 58)		After intervention (n = 58)	
			Number of cases	Percentage (%)	Number of cases	Percentage (%)
SAS	severe	≥70	23	37.9	2	3.4
	moderate	60‐69	24	43.1	29	50.0
	mild	50‐59	10	17.2	24	41.4
	None	<50	1	1.7	3	5.2
SDS	severe	>72	20	34.5	6	10.3
	moderate	63‐72	35	62.1	43	74.1
	mild	53‐62	3	3.4	9	15.5
	none	≤52	0	0	0	0

## 4 DISCUSSION

### 4.1 Psychological intervention for head and neck cancer patients undergoing radiotherapy during COVID‐19 outbreak

In this research, *Guidelines of Psychological Adjustment During Novel Coronavirus Outbreak* was released by National Health Commission of the People's Republic of China on February 2020 which was used as the primary source in this article. *Clinical Practice Guidelines in Oncology*, *Version 1*, *2020* published by the US National Comprehensive Cancer Network was used as supplementary guidelines. By combining both guidelines, a comprehensive psychological intervention strategy was developed.^8^, ^9^ As indicated in the *Psychological Intervention Guidelines During Novel Coronavirus Outbreak*, the main approaches for psychological intervention during the outbreak include internet‐based intervention and face‐to‐face intervention.^10^

Based on actual conditions during the outbreak, psychological intervention for patients utilized internet‐based intervention (online WeChat communication channel) and face‐to‐face intervention (offline one‐to‐one interviews). Patients involved in this research mainly received psychological intervention from WeChat communication channel; this was chosen for the advantage of flexible location and time options achieved by instant and effective online communication. At the same time, internet‐based intervention (online WeChat communication channel) helps to protect medical staff and patients from being infected during social interaction. Offline face‐to‐face interview was used as a supplementary psychological intervention approach. Patients were encouraged, under confidentially, to express their concerns and worries to the research assistants as to prevent negatively affecting the emotional stability of other patients in this research. Also, one‐to‐one interviews promote the establishment of relationships between patients and medical staff.

Research shows accurate and reliable information sharing facilitates the alleviation of a sense of panic and loss caused by lack of information.^17^ Appropriate social activities have shown to promote the secretion of noradrenaline and 5‐hydroxytryptamine, regulate signals of limbic system, reduce excessive secretion of cortisol, and ameliorate emotional disorders.^18^ Recalling past positive personal experiences can help patients increase their adaptability to the current situation, reduce the level of anxiety and depression, and improve their overall quality of life.^19^, ^20^, ^21^, ^22^, ^23^, ^24^, ^25^ Encouraging patients to vent their negative emotions can help them to achieve catharsis and thus relieving psychological pressure.^26^

### 4.2 Psychological state of cancer patients undergoing radiotherapy during the Covid‐19 outbreak

Patients enrolled in this research were all with newly diagnosed head and neck cancer (including stages II, III, and IV at the point of diagnosis). All of the patients were in the process of undergoing 6 to 7 weeks of radiotherapy treatment with total doses of 69 to 72 Gy.

Through a comparison with National norm of SCL‐90, scores corresponding to the nine dimensions (somatization, obsessive, interpersonal sensitivity, depression, anxiety, hostility, phobic anxiety, paranoid ideation, and psychoticism) of the patients were observed to be higher than the scores of the national norm (Table 2). Increased scores may be directly correlated with cancer treatment, the cost of treatment, cancer symptoms, side effects caused by cancer treatment, concerns about prognosis, the new hospitalization environment, and the stress response to the sudden outbreak of COVID‐19. Studies showed that cancer patients could show higher scores than that of the healthy population in the nine dimensions.^27^, ^28^ In addition, in agitation inducing phenomena, the general population is shown to display symptoms of somatization, anxiety, and depression.^24^ Similar results were observed in this research.

By comparing scores corresponding to the dimensions of SCL‐90, patients with head and neck cancer receiving psychological intervention exhibit a reduction in overall average scores according to all nine dimensions. Results showed that psychological state of patients was significantly improved in each dimension (*P* < .05) after undergoing psychological intervention (Table 2). However, the scores are still higher than the level of the national norm, which could be contributed to by symptoms related to cancer treatment, financial burden caused by cancer, concerns on the prognosis, and the new hospitalization environment.

### 4.3 Anxiety and depression of head and neck cancer patients undergoing radiotherapy during Covid‐19 outbreak

Domestic and international studies indicated that distinct groups of people including infected populations, medical staff, older adults, and populations with underlying medical conditions suffered from various neuroses during the period of SARS outbreak, predominant among these disorders is depression and anxiety.^29^, ^30^, ^31^ Customized psychological intervention was shown to improve the overall psychological state of cancer patients undergoing radiotherapy during COVID‐19 Outbreak, especially with the factors of anxiety and depression. As shown in Table 4, severity of the patient's depression and anxiety were significantly lowered after psychological intervention. As for the aspect of anxiety before psychological intervention, the detection rate of severe anxiety and moderate anxiety was 81%. After psychological intervention, the detection rate of severe anxiety and moderate anxiety decreased to 53.4%. In terms of depression, the detection rate of severe depression decreased from 34.5% to 10.3% after psychological intervention. This result is consistent with other similar research.^21^, ^22^, ^25^, ^26^

## 5 LIMITATION

Our study has some potential limitations. First, it was not a randomized controlled trial. Second, patients enrolled in the research are limited to head and neck cancer patients undergoing radiotherapy which could not represent all cancer patients and cause potential sampling errors. Third, patients enrolled were at different clinical stages which meant we were unable to determine if the stage of cancer affect outcomes of psychological intervention. Fourth, small sample size and short follow‐up time perhaps creating bias.

## 6 CONCLUSION

Local or systemic metastatic cancer is characterized by its long course and severity. Therefore, cancer patients are at high‐risk for severe illness from COVID‐19; they are more likely to develop severe complications and multiple organ failures which lead to high mortality rates.^32^ Thus, cancer patients, as a susceptible population, tend to suffer more from psychological distress in the event of public health crises.^33^ Under premise of implementing protective measures during the outbreak, multi‐level, multi‐channel, and diversified psychological intervention measures should be carried out proactively for cancer patients as early as possible.^34^ Furthermore, it is critical to prioritize the minimization of psychologically adverse effects caused by an outbreak on cancer patients with the objective of achieving mental stability, a positive psychological state, a reduction of depression, anxiety, and other negative emotions. It has been proven that, for cancer survival, social isolation has a significant impact and it is necessary to maintain patients' social infrastructure as much as possible.^35^, ^36^ For cancer patients, psychological intervention is recommended, and it is critical during the outbreak for hospitals and medical staff to employ developed systems assisting in maintaining patients as psychologically stable throughout the outbreak.^37^ Currently, many countries have established theories about psychological intervention against public health events, but there is a lack of unified technical standards.^38^, ^39^ In the future, we need to increase the number of samples and follow‐up from time to time to conduct more in‐depth, detailed research.

## CONFLICT OF INTEREST

The authors declare that they have no conflict of interest regarding this article.

## AUTHOR CONTRIBUTIONS

Dejing Xu contributed to the concept of the study; Shuchang Lou contributed to contributed significantly to analysis and wrote the manuscript; Xiaodong Li and Yan Huan performed the experiment and data analysis; Jun Li helped the design of the experiment with constructive discussion and instruction.

## ETHICS STATEMENT

The study was approved by the Jiangsu Cancer Hospital Ethics Committee and all patients gave written informed consent.

## References

[1] Chen DM , Zhao XQ , Miao YG , et al. Analysis of the global coronavirus related research status and its enlightenment for the present and future. Chinese J Clin Med. 2020;27(1):1‐12.

[2] Wang K , Kang SR , Tian RH , Wang Y , Zhang XZ , Li HM . CT characteristic appearances of patients with novel coronavirus pneumonia. Chinese J Clin Med. 2020;27(1):27–31.

[3] Na Z , Dingyu Z , Wenling W , et al. A novel coronavirus from patients with pneumonia in China, 2019. The New England Journal of Medicine. 2020;382:727‐733. 31978945 10.1056/NEJMoa2001017 PMC7092803

[4] Abad C , Fearday A , Safdar N . Adverse effects of isolation in hospitalised patients: a systematic review. Journal of Hospital Infection. 2010;76(2):97‐102. 10.1016/j.jhin.2010.04.027 PMC7114657 20619929

[5] Peters S , Karin J , Brandt J , et al. Representatives of the European Society for Medical Oncology (ESMO). https://www.esmo.org/for-patients/patient-guides/cancer-care-during-the-covid-19-pandemic. Accessed April 18, 2020.

[6] Coronavirus: What People with Cancer Should Know. National Caner Institute. https://www.cancer.gov/contact/emergency-preparedness/coronavirus#if-i-have-cancer-now-or-had-it-in-the-past-how-can-i-protect-myself. Accessed April 30, 2020.

[7] Humphris G . Psychological management for head and neck cancer patients: United Kingdom National Multidisciplinary Guidelines. J Laryngol Otol. 2016;130(S2):S45‐S48. 10.1017/S0022215116000426 PMC4873900 27841113

[8] National Health Commission of the People's Republic of China , . Guidelines of Psychological Adjustment During Novel Coronavirus Outbreak. http://www.gov.cn/fuwu/2020-02/08/content_5476190.htm. Accessed April 18, 2020.

[9] Michelle BR , Kristine AD , Kauser A , et al. NCCN Clinical Practice Guidelines in Oncology, Version 1.2020. Journal of the National Comprehensive Cancer Network. https://www.nccn.org/professionals /physician_gls/default.aspx. Accessed April 10, 2020.

[10] Zhen YL , Lou YZ . Psychological Intervention Guidelines During Novel Coronavirus Outbreak http://m.zhangyue.com/readbook/12127790/2?p2=116813. Accessed April 10, 2020.

[11] Bonicatto S , Dew MA , Soria JJ , Seghezzo ME . Validity and reliability of symptom checklist 90 (SCL90) in an argentine population sample. Social Psychiatry Psychiat Epidemiol. 1997;32:332‐338. 10.1007/BF00805438 9299927

[12] Xie H , Dai HQ . Evaluation of SCL‐90 checklist. Nervous Disease Mental Health. 2006;02:156‐159.

[13] Wang XD , Wang XL , Ma H . Mental health evaluation handbook. Chinese Mental Health J. 1999;04:31‐35.

[14] Jin H , Wu WY , Zhang MY . SCL‐90 preliminary results and analysis of Chinses healthy people. Chinese J Nervous Mental Dis. 1986;12(5):260‐262.

[15] Ramirez SZ , Lukenbill JF . Psychometric properties of the Zung self‐rating anxiety scale for adults with intellectual disabilities (SAS‐ID). J Develop Phys Disabilities. 2008;20:573‐580.

[16] Jegede RO . Psychometric properties of the self‐rating depression scale (SDS). J Psychol. 2010;93(1):27‐30. 10.1080/00223980.1976.9921370 1003334

[17] Su L , Wei B . Psychological reactions and interventions during emergent events of public health. Chinese J Behav Med Sci. 2005;14(12):1139‐1141.

[18] Martinsen EW . Physical activity and depression: clinical experience. Acta Psychiat Scand. 1994;377(377):23‐27. 10.1111/j.1600-0447.1994.tb05797.x 8053362

[19] Lyu QY , Cong Z . Psychological interventions during the outbreak of SARS. Chinese Mental Health J. 2003;17(8):534‐535.

[20] Shi GR , Li DM , Wang L , et al. An investigation on psychological state and interventions for inpatients with traumatic fracture. J Nurses Train. 2006;21(1):21‐23.

[21] Tang HY , Wang CG , Qiu ZJ . Effects of psychological interventions on the mental health and happiness of patients with infertility. Maternal Child Health Care of China. 2016;31(17):3579‐3581.

[22] Dai BR , Gao J , Yuan Q , He GP , Feng H . Study on intervention effect of group reminiscence therapy on self‐esteem and emotional balance of the elderly in community. Chinese Nursing Res. 2010;24(19):1704‐1707.

[23] McCloskey JC , Bulechek GM . Nursing Interventions Classification (NIC). 2nd ed. St.Louis: Mosby; 1996:38‐41. 8591448

[24] Hsu Y‐C , Wang J‐J . Physical, affective, and behavioral effects of group reminiscence on depressed institutionalized eldersin Tai‐wan. Nursing Res. 2009;58(4):294‐299. 10.1097/NNR.0b013e3181a308ee 19609181

[25] Tsang HW , Fung KM . A review on neurobiological and psychological mechanisms underlying the anti‐depressive effect of qigong exercise. J Health Psychol. 2008;13(7):857‐863. 18809635 10.1177/1359105308095057

[26] Tan JR , Luo X , Lu SY , et al. Intervention effect of emotional catharsis on patients with bipolar disorder. J Clin Nursing's Pract. 2018;3(20):110‐111, 114.

[27] Pan XF , Fei MD , Zhang KY , Fan ZL , Fu FH , Fan JH . Psychopathological profile of women with breast cancer based on the symptom checklist‐90‐R. Asian Pacific J Cancer Prevent. 2014;14(11):6579‐6584. 10.7314/apjcp.2013.14.11.6579. 24377571

[28] Fafouti M , Paparrigopoulos T , Zervas Y , et al. Depression, anxiety and general psychopathology in breast cancer patients: a cross‐sectional control study. In Vivo. 2010;24(5):803‐810. 20952755

[29] Maunder R , Hunter J , Vincent L , et al. The immediate psychological and occupational impact of the 2003 SARS outbreak in a teaching hospital. Canad Med Association J. 2003;168:1245‐1251. PMC154178 12743065

[30] Xiao JQ , Wu QH , Hao YH , et al. Investigate Psychological Situation of the Harbin People During SARS, Analysis its Influencing Factors and Intervention Strategy. Chinese Health Economics, 2007;26(3):20‐23.

[31] Lu L , Liang ZQ , Xu Y , Zhang KR . Investigation on Psychosomatic Symptoms of SARS Patients at Different Stages. China Preventive Medicine, 2008;04:268‐ 270.

[32] Zhang L , Zhu F , Xie L , et al. Clinical characteristics of COVID‐19‐infected cancer patients: a retrospective case study in three hospitals within Wuhan, China. Annals Oncol. 2020;31:894‐901. 10.1016/j.annonc.2020.03.296 PMC7270947 32224151

[33] Shuman AG , Pentz RD . Cancer research ethics and COVID‐19. Oncologist. 2020;25:1‐2. 32227551 10.1634/theoncologist.2020-0221 PMC7228207

[34] Kang C , Yang S , Yuan J , Xu L , Zhao X , Yang J . Patients with chronic illness urgently need integrated physical and psychological care during the COVID‐19 outbreak. Asian J Psychiatry. 2020;2020:51. 10.1016/j.ajp.2020.102081 PMC7139251 32289729

[35] Moore S , Leung B , Bates A , Ho C . Social isolation: impact on treatment and survival in patients with advanced cancer. J Clin Oncol. 2018;36:156.

[36] Kotronoulas G , Papadopoulou C , Burns‐Cunningham K , Simpson M , Maguire R . A systematic review of the supportive care needs of people living with and beyond cancer of the colon and/or rectum. Eur J Oncol Nurs. 2017;29:60‐70. 28720267 10.1016/j.ejon.2017.05.004

[37] Jiao JY , Hu Z , He CS , Yang P , Fang L . Advances in psychological crisis intervention during public health emergencies. Med Soc. 2014;27(3):78‐81.

[38] Morgan DJ , Diekema DJ , Sepkowitz K , Perencevich EN . Adverse outcomes associated with contact precautions: a review of the literature. Am J Infect Control. 2009;37:85‐93. 19249637 10.1016/j.ajic.2008.04.257 PMC3557494

[39] Ding SR , Shi J , Wang TL , Wang BH . An investigation of public mental health during SARS outbreak. Chinese J Public Health. 2005;21(9):1119‐1120.

